# Dr. Spalding on the Portsmouth Bill of Mortality

**Published:** 1812-07

**Authors:** L. Spalding

**Affiliations:** Portsmouth, New Hampshire


*To the Editors of the Medical and Physical Journal.*

GENTLEMEN,

I HAVE inclosed the Portsmouth Bill of Mortality for 1810-1, and the Fairfield Catalogue.*

The bills for the three last years are constructed differently from those heretofore published. They shew the disease, age, and sex, of each individual, as well as the month in which death took place; and, moreover, what, so far as has come to my knowledge, has never been introduced before, i.e. whether the subject has been married or not. This addition was made in consequence of the following query having been made, by my friends Professors Rush and Barton, of Philadelphia, in their public lectures—"Whether the marriage state is conducive to longevity?"

Judge Meyer, of New York, has lately published a treatise on "Insurance on Lives and Life Annuities," in which he has quoted the Portsmouth bills, making certain estimates of the probable duration of life therefrom.

I have the honor to be, Gentlemen,

Your's, &c.

L. SPALDING.

*Portsmouth, New Hampshire, Feb.* 12, 1812.

---

* Vide Intelligence.

### *Bill of Mortality for Portsmouth, New Hampshire, for A.D.* 1810.

| *Complaint.* | *Age.* | *Jan.* | *Feb.* | *March* | *April* | *May* | *June* | *July* | *August* | *Sept.* | *Oct.* | *Nov.* | *Dec.* | *Total* |
|---|---|---|---|---|---|---|---|---|---|---|---|---|---|---|
| Apoplexy . . . . | 78‘ years | | 1 | | | | | | | | | | | 1 |
| Atrophy | 1, w 76‘ y 3. d 2, 5. 68‘ y 2, m 2, 47‘ y 10. d 4. m 56, 2 y | 1 | 1 | 3 | | 2 | | 1 | | 3 | | 1 | 1 | 13 |
| Chlorosis . . . . | 13, years | | | | | | | | 1 | | | | | 1 |
| Cholera of infants | 4 m 2. 2, y 1, m 1. 1. 1, year | | | | | | | | 1 | 2 | 2 | 2 | | 7 |
| Consumption | 33. 55‘ 36. 23, 63‘ 35. 64‘ 58, 20, 21. 42. 37‘ 25‘ 52‘ 69‘ 40‘ 15. 41‘ 38‘ 41‘ 23. 28, 17, 25‘ 44‘ 28‘ 34‘ years | | 2 | 4 | 2 | 1 | 3 | 3 | 4 | 1 | 4 | 1 | 2 | 27 |
| Convulsions | 5. m 2, y 6. m 4. years | | | 2 | | | | | 1 | | | | 1 | 4 |
| Croup . . . . . . . | 2. years | | | | | | | | | | | | 1 | 1 |
| Dropsy . . . | 40‘ 60‘ 2. years | | | | | | 1 | | 1 | | | 1 | | 3 |
| Dropsy in the brain | 2. 13, y 5. m 2. 5, 4, 2, y 3, months | | 1 | 1 | 1 | 1 | | 1 | | 1 | 1 | | 1 | 8 |
| Erythema . . | 3. m 1, week | | 1 | | | | 1 | | | | | | | 2 |
| Fever, inflammatory . | 5. 4, years | | | | | | 1 | | 1 | | | | | 2 |
| Fever, petechial . . | 9, years | | | | 1 | | | | | | | | | 1 |
| Fever, puerperal . | 18‘ 44‘ years | | | 1 | | | | | | | | | 1 | 2 |
| Fever, pulmonic | 52‘ 75‘ 78‘ 5, y 4, m | 1 | 2 | | 1 | | | | 1 | | | | | 5 |
| Gout . . . | 74‘ years | | | | | | | | | 1 | | | | 1 |
| Hæmorrhage . | 69‘ 52‘ years | | 1 | | | | | | 1 | | | | | 2 |
| Hooping cough . | 1, 3, years | | | | | | | | | | 2 | | | 2 |
| Inflammation . . | 46‘ years | | | | | 1 | | | | | | | | 1 |
| Mortification . | 53‘ 67‘ years | | | | | 1 | | 1 | | | | | | 2 |
| Old age | 89‘ 90‘ 77‘ 89‘ 90‘ 79‘ 78‘ y | 2 | 1 | | 2 | | | 2 | | | | | | 7 |
| Palsy . . | 58‘ 74‘ 87‘ 60‘ years | 1 | | 1 | | | | | | | 2 | | | 4 |
| Phrenitis . . . | 50‘ years | | | 1 | | | | | | | | | | 1 |
| Quinsy . . | 7. m 1. 1, year | 2 | | | | | | | | 1 | | | | 3 |
| Scirrhus liver . . | 25. years | | | | | | | 1 | | | | | | 1 |
| *Casualties.* Burnt . . | 75‘ 4, 1, year | | | | | | | | | | 1 | 2 | | 3 |
| *Casualties.* Drowned . . | 2. 7. years | | | | | | | 1 | | | 1 | | | 2 |
| *Casualties.* Overlaid . . | 2. months | | | | | | | | | | | 1 | | 1 |
| *Casualties.* Run over by a waggon | 45‘ 5. y | | | | 1 | | | 1 | | | | | | 2 |
| *Casualties.* Suicide . . | 19. 25‘ years | | | | 1 | | | | | | | 1 | | 2 |
| *Births* {Males 130, Females 122} 252; Still-born 6. | | | | | | | | | | | | | | |
| *Marriages* 64. | Total | 7 | 10 | 13 | 9 | 6 | 6 | 11 | 11 | 9 | 13 | 9 | 7 | 111 |

The month of January exhibited the coldest, as well as the most remarkable, change of weather ever recorded in New Hampshire. At twelve o'clock at noon of the 18th the thermometer was at 42°, and at twelve the 19th it had fallen 12° below zero. It fluctuated between 7° and 14° below zero from the 19th to the 22d. We do not find that either this sudden change, or extreme cold weather, had any material effect on the health of our citizens.

*Note.*—This bill is so constructed as to show the complaint, age, sex, &c. and whether married or unmarried. When a period follows the age, it denotes the male sex; a comma the female sex; when in their usual place at the bottom of the line, unmarried—at the top of the line, married.

***Bill of Mortality for Portsmouth, New Hampshire, for A.D.* 1811.**

| *Complaint.* | *Age.* | *Jan.* | *Feb.* | *March* | *April* | *May* | *June* | *July* | *August* | *Sept.* | *Oct.* | *Nov.* | *Dec.* | *Total* |
|---|---|---|---|---|---|---|---|---|---|---|---|---|---|---|
| Angina Pectoris . . | 58· years | | | | | | | | | | | | 1 | 1 |
| Aphtha . . | 1. month | | | | | | | 1 | | | | | | 1 |
| Apoplexy . . . . | 75‘ years | | 1 | | | | | | | | | | | 1 |
| Atrophy | 2. m 4. 7. 68, 64, 7, 2. 3. y | | 2 | 1 | 1 | 1 | | 1 | | | 1 | 1 | | 8 |
| Cholera of infants | 1, 2. years | | | | | | | | | | 1 | | 1 | 2 |
| Colic, bilious . . | 17. years | | | | | | | 1 | | | | | | 1 |
| Consumption | 29‘ 60· 45‘ 35‘ 20. 48, 30‘ 59‘ 25· 48· 40· 33‘ 68‘ 69; 25· 30· 20. 70. 32· 44· 35‘ 60· 65· 54, 81· 30‘ years | 2 | 1 | 1 | 2 | 2 | 2 | 5 | 3 | 1 | 2 | 4 | 1 | 26 |
| Convulsions | 1. y 3, d 3. w 6. y 2. 3. 1, w 2. y 2. m 1. w 1. m 1. d | | 2 | 1 | | | 1 | 1 | 2 | | 2 | 2 | 1 | 12 |
| Cynanche maligna . . | 3. years | | | | | 1 | | | | | | | | 1 |
| Diseased vertebræ . | 24, years | | | | | | | | 1 | | | | | 1 |
| Dropsy . . | 64· 60‘ 68‘ 31‘ years | 1 | | 1 | | | | 1 | 1 | | | | | 4 |
| Dropsy in the brain | 3. 2, y 2. 3, m 14. 4. 1. 1, year | | | 1 | | | | | 2 | 2 | 2 | | 1 | 8 |
| Erythema . . | 2. 3, months | | | | | 1 | | | | | | 1 | | 2 |
| Fever, pulmonic . . | 2. years | | | | | | 1 | | | | | | | 1 |
| Hæmorrhage . | 28‘ 35‘ 69‘ years | | 1 | | | | 1 | | | | | | 1 | 3 |
| Hooping cough . . | 4. years | | | | | | | | | | | 1 | | 1 |
| Intoxication . . | 31· years | | | | | | | | 1 | | | | | 1 |
| Mortification | 1, 48, 2, 90, 4, years | | 1 | | | | | 1 | | | 1 | 1 | 1 | 5 |
| Old age | 87· 83‘ 70‘ 76‘ 90, 87· 74‘ 81· 80, 88· 90· 85· years | | 1 | 1 | 1 | 1 | 1 | | 2 | 1 | 1 | 1 | 2 | 12 |
| Palsy . . | 69. 78‘ 58‘ 72‘ years | | | 2 | 1 | | | | | | | | 1 | 4 |
| Phrenitis . . | 30· 8, years | | | | | 1 | | | | | 1 | | | 2 |
| Quinsy . . . . | 2, years | 1 | | | | | | | | | | | | 1 |
| Rheumatism . . . | 55· years | | 1 | | | | | | | | | | | 1 |
| Scrophula . . | 26, 10, years | | | | | 1 | | | | | 1 | | | 2 |
| Small-pox . . | 20. years | | | | | | 1 | | | | | | | 1 |
| Tetanus . . . | 14. years | | | | | | | 1 | | | | | | 1 |
| *Casualties.* Drowned | 78‘ 62· 50· 40. y | | | | | | 1 | 1 | | | | 2 | | 4 |
| *Casualties.* Falling of earth | 35· years | | | | | | | | | 1 | | | | 1 |
| *Casualties.* Suicide · | 18, 41. years | | | | 1 | | | 1 | | | | | | 2 |
| *Births* { Males 130, Females 124 } 254 | | | | | | | | | | | | | | |
| Still-born 15. | | | | | | | | | | | | | | |
| *Marriages* 69. | Total | 4 | 10 | 8 | 6 | 8 | 8 | 14 | 12 | 5 | 12 | 13 | 10 | 110 |

